# “It’s More Than Just Exercise”: Tailored Exercise at a Community-Based Activity Center as a *Liminal Space* along the Road to Mental Health Recovery and Citizenship

**DOI:** 10.3390/ijerph181910516

**Published:** 2021-10-07

**Authors:** Kjersti Karoline Danielsen, May Helena Øydna, Sofia Strömmer, Kenneth Haugjord

**Affiliations:** 1 Department of Nutrition and Public Health, Faculty of Health and Sport Sciences, University in Agder, 4604 Kristiansand, Norway; 2 Unit for Decision and Coordination of Services, Department of Health and Care, 4611 Kristiansand, Norway; may.helena@oydna.net; 3 MRC Lifecourse Epidemiology Unit, Southampton General Hospital, University of Southampton, Tremona Road, Southampton SO16 6YD, UK; ss3@mrc.soton.ac.uk; 4 District Psychiatric Center Østre Agder, Clinic for Mental Health and Addiction, Sørlandet Hospital, 4824 Bjorbekk, Norway; kenneth.haugjord@sshf.no

**Keywords:** mental health challenges, physical activity, exercise, community-based activity center, recovery, citizenship, qualitative method

## Abstract

Mental health care policies call for health-promoting and recovery-oriented interventions, as well as community-based programs supporting healthier habits. The purpose of this study was to explore how individuals facing mental health challenges experienced participating in tailored exercise at a community-based activity center, and what role tailored exercise could play in supporting an individual’s process of recovery. Data were collected through in-depth interviews with nine adults experiencing poor mental health who engaged in exercise at the activity center. Interviews were audio-recorded, transcribed verbatim and analyzed using systematic text condensation. Participants spoke about the community-based program being a safe space where they could “come as they are” (Theme 1). Taking part in the program was “more than just exercise” and allowed them to connect with others (Theme 2). The experiences they gained from exercise also helped with other areas in life and provided them with a safe space to build their confidence towards the “transition back to the outside” (Theme 3). We summarized the findings into one overall theme: “inside vs. outside”. In conclusion, a community-based activity center acted as a liminal space that aided mental health recovery by allowing participants to feel safe, accepted and supported, as well as experience citizenship. The findings highlight the need to treat mental health challenges as a contextual phenomenon and creating arenas for community and citizenship in society.

## 1. Introduction

Most people experience mental health challenges during their lives, either by facing a mental health challenge themselves or by family members or friends being affected. According to the WHO, the burden of mental health challenges is growing, with tremendous impacts on health as well as major social and economic consequences in all countries of the world [1].

When mental health challenges arise, the condition affects the whole person and is reflected in the person’s thoughts, feelings, behavior, and social interactions with other people [1,2]. Thus, facing mental health challenges has profound impacts on many domains in life, such as impaired functioning in everyday life and reduced quality of life, as well as dropping out of school, studies and working life [2,3,4]. Several recovery studies also point out the impact on identity, the loss of positive roles and several negative effects of the stigma associated with mental health challenges, such as difficulties in finding work [5,6]. In the opposite direction, low education, being outside work and poor finances are important contributors to mental health challenges [3,7].

In all societies there are human experiences with mental health challenges, and it can be understood as not just a private experience but something that is part of a social and cultural context [8]. Research at a population level would suggest that social factors rather than medical interventions are the main determinants of recovery from mental health challenges [9,10]. As mental health challenges arise in the context and society that human beings live in, mental health recovery is expected to happen in psychosocial contexts in society [2,11]. The United Nations (UN) report, “Right to mental health” [12] also calls for a change in focus, aiming for mental health challenges to be treated as contextual phenomenon. This is, however, not yet reflected within the support systems, which can still be dominated by traditionally biomedical models where mental health challenges are reduced to phenomena more or less disconnected from the context where they occur and in simplified relations to life in general [13,14]. 

### 1.1. Physical Activity

Nevertheless, treatment is one route among many to recovery [15]. Given a limited capacity to assist everyone who seeks help for mental health challenges, there is a need for simple and easily accessible strategies people can use themselves to better manage mental health and recover [4,16,17]. Physical activity shows potential in this respect and can be implemented/carried out at a community-based activity center. 

In recent years physical activity has been recognized as an important area of responsibility in public health policy and is included in plans and strategies. The importance of physical activity as part of mental health work is at the center of the Norwegian Government’s latest strategic plan for better mental health [18]. According to the latest public health report in Norway, there is a special need for efforts to offer people with mental health challenges help to change their lifestyle [19]. National and international mental health care policies also call for health-promoting recovery-oriented interventions, as well as community-based programs supporting healthier habits [2]. 

A large amount of quantitative research has shown associations between physical activity and mental health [20,21,22]. In accordance with the well-known postulated psychological effects of physical activity, participants in several qualitative studies report a positive impact on mental health, improvements in symptoms of mental health illness and better management of the illness/symptoms through physical activity [23,24,25,26,27,28]. Many of these studies on physical activity and mental health assume a connection between physical and mental health through some physiological and endocrine changes and focus on the impact of physical activity on the symptoms of mental disorders [20,21,29,30,31]. There has been less focus on the aspect of physical activity as something that can facilitate other processes associated with recovery and a better quality of life and well-being.

### 1.2. Recovery

Recovery is a term used, understood and defined in different ways [32,33,34,35], and was recently defined by Topor, Larsen [35] to be “*a deeply social, unique and shared process in which our living conditions, material surroundings, attitudes, values, feelings, skills, and/or roles are changing. It is a way of living satisfying lives together with others, even though we may still experience distress, unusual experiences, and troubling behavior. Recovery involves engaging in new material and social contexts and in dialogues where new ways of understanding and handling the situation are created as we move beyond the psycho-social-material crisis*”. This definition is like recovery itself: a process. Recovery from mental health problems is not an outcome to arrive, it is a journey into life, a personal and social journey [36,37]. Common to all definitions is that recovery does not imply that the mental health challenges or illness are cured. The main aspect is being able to live meaningful and satisfying lives, as the people themselves define it, with or without symptoms and problems that will come and go [2,38,39,40,41]. Disease and symptom focus is toned down whilst humanization, participation, mastery and citizenship will receive increased focus. In line with this, recovery and the knowledge about recovery relies on people’s own experiences of facing mental health challenges and how such problems affect everyday life. These lived experiences guide our understanding of what might be important in such recovery processes [2,11,42,43].

In studies exploring the experiences of what people facing mental health challenges think is most important for mental health and recovery, five dimensions recur; (1) connectedness, (2) hope and optimism about the future, (3) identity, (4) meaning in life, and (5) empowerment (abbreviated CHIME) [42,44]. Recovery-oriented mental health work proposes that processes related to CHIME mainly take place outside the health services; it is about everyday life in relationships and interaction with others in the local environment/community, it is about a recovery process throughout the lifespan [2]. This can involve helping people who have mental health challenges in facilitating these processes and supporting the individual in their recovery process. Research has shown that physical activity may provide an opportunity for social interaction [23,25,27,28,45,46,47,48,49,50]; give a sense of meaning, purpose and achievement [23,24,25,27,28,45,46,48,50,51]; as well as being a useful self-help strategy and a source for regaining life [26,46,51,52]. Facilitating social interaction with other people through exercise, is also shown to contribute to hope and optimism for the future, as well as developing a new positive identity other than mental health challenges [35]. In a literature review including thirteen qualitative studies on exercise interventions for people with mental health challenges, Mason and Holt [28] found that service users experience exercise interventions as inclusive, non-stigmatizing and effective for their personal recovery process. People facing mental health challenges also emphasize facilitating personnel [23,25,53,54].

### 1.3. Citizenship

As there is a growing body of empirical data that reveals that recovery has to be viewed as a social and relational process, recovery is also an emancipation movement that seeks the social inclusion of people who have been marginalized and segregated [40]. As such, promoting citizenship is a key characteristic/domain of recovery-oriented practice [55]. Citizenship is an approach to supporting social inclusion and participation in society of people with mental illnesses [56]. Recovery and citizenship evolved independently, and there are both differences and similarities between the two approaches [56]. As stated by Ponce and Rowe [56], both recovery and citizenship have an expansive view of the person, refusing to define individuals by disability but rather as people first who, among other characteristics and roles (e.g., sibling, parent, worker, musician, and neighbor), are facing mental health challenges. Nevertheless, as recovery validates the individual’s hopes and strivings in the social context, citizenship adds a social-contextual emphasis and involves the strength of people’s connections to the rights, responsibilities, roles, resources and relationships (the five Rs) that society offers to people through public and social institutions and associational life, and “a sense of belonging as full, participating members in society that is validated by one’s fellow citizens” [57,58]. In Norway and other countries, studies have shown that street football can be an important arena for recovery processes among people facing mental health challenges [59]. This research has its origins across services and systems, as well as a focus on the users being involved and considered equal parts/contributors. The football training facilitates the participant’s recovery processes through authority and confirmation of one’s own resources and motivation, providing a sense of belonging as well as being an arena for social training with active participation and co-determination [59].

### 1.4. Aim of the Study

To the best of our knowledge, the current body of qualitative research exploring the experiences of physical activity among people facing mental health challenges, does not cover tailored exercise at community-based activity centers. Increased knowledge about such centers is important to further develop and improve them. Those who can contribute to the knowledge in the best possible way are the participants themselves. By understanding people’s experiences, we can identify their key needs, which will help design more effective support programs/facilities and reduce national health service costs. Consequently/within this context, the purpose of this study was to explore how individuals facing mental health challenges experience participation in tailored exercise at a community-based activity center, and what role tailored exercise could play in supporting an individual’s process of recovery. 

## 2. Materials and Methods

### 2.1. Study Design

The study was designed as a qualitative explorative study using individual, semi-structured, in-depth interviews [60,61]. An inductive, descriptive, and explorative type of analysis was used, i.e., systematic text condensation developed by Kirsti Malterud [60,62]. 

### 2.2. Research Team

In this study, the research team consisted of an associate professor in sports science and public health (first author), a master’s student in psychosocial health, a psychologist specialist in community psychology, working as a professional advisor and service developer at the district psychiatric center (DPS) at the Hospital of Southern Norway, and a psychologist and researcher in behavioral science, specializing in motivation (co-authors). To increase the validity and reliability of the project, the study, with its analyses and findings were also discussed within the research group “An including society” at the University of Agder (webpage: https://www.uia.no/en/research/helse-og-idrettsvitenskap/an-including-society (accessed on 6 October 2021)). In this group, there is a person with user experience of facing mental health challenges and experience of the importance of physical activity for his own recovery process. This person has contributed to the design of the interview guide and the study. None of the members of the research team or the group work at the activity center.

### 2.3. Preconceptions

The aspect of researchers’ preconceptions is highlighted by Malterud [60], Malterud [62]. The motivation for this study arose from the master’s student’s experiences during a practice period at the activity center, and from the knowledge of the quantifiable effects of physical activity in the treatment of mental health challenges. We reflected on our own background and preconceptions, both prior to and during the research process, and how they could affect the prepared research questions and our way of understanding the participants’ descriptions of their experiences. This helped us to be aware of and understand our preconceptions as a resource for new questions during the interview and created an informed platform to understand our participants in new ways. We tried to bracket our preconceptions [60,62]. During the interviews, we attempted to set aside the preconception through bracketing interviews [60]. This is to be able to critically draw attention to the participants’ experiences in light of the individual’s lifeworld (Malterud, 2018, p. 29). Nevertheless, our background and pre-understanding is a necessary condition for our understanding, and we acknowledge that as a researcher we are always “visible” as acting in the themes and text [60,62,63]. We aimed to describe the participants’ experiences with physical activity at the activity center as accurately as possible, keeping in mind Giorgi’s statement that “the result of a descriptive analysis is a second-order description” [64]. The analysis/findings were also discussed within the research group “An including society”.

### 2.4. Research Context and Tailored Exercise

The research context is a community-based activity center located in a larger municipality in southern Norway for people aged 18 years or more facing mental health challenges or substance abuse. The center has a “non-clinical approach”, i.e., there is no requirement for proven diagnosis and no referral is needed. As there is no waiting list, participants can enter the activity center free of charge at any time for as long as they want, and participants are referred to as members. However, on their first visits to the center many of the participants are accompanied by a “follower”, such as a mental health worker in the municipality, the hospital, or the district psychiatric center. The municipality finances the center, and for trips and larger excursions, it is also supported by scholarships, funds, etc.

A large variety of activities, groups and courses is offered at the center, including outdoor life, training and movement, creative activities, choir and music and various mastery and development groups. The focus is on opportunities, joy, activity, and community. In addition, there is a popular coffee shop with reasonable prices and open events to attend. 

The activity center focuses on the services being recovery supportive, meaning that recovery is a basic attitude in their meetings with the participants and the employees work to support the participants in their personal and social recovery process. The aim of the activity center is for everyone in the target group to have the opportunity to live active lives, participate in meaningful activities as well as be part of a good social community. A recovery-oriented approach to all participants is implemented by focusing on the participants’ goals and opportunities and being concerned about what is important to the participants. The participants are actively involved and have an influence on which groups and activities should be carried out.

A key part of the offer at the activity center is the tailored exercise. The exercise sessions are supervised by personnel with competence within sports science and mental health. The participants are introduced to exercise tailored to their mental health, physical fitness and function, and exercise together with other individuals with mental health challenges. The exercise includes endurance training (in the form of running classes and spinning) and strength training sessions (in the form of CrossFit and Tabata). Participants can also exercise with a personal coach. The intention is to present what is possible for the participants and the experience of mastering and enjoying being physically active.

### 2.5. Participants and Recruitment

The participants were five women and four men (aged 20–45 years). All the participants had voluntarily entered the center and wanted to attend the exercise sessions at the activity center for people facing mental health challenges. As the center has a “non-clinical approach”, and is a low threshold offer, the participants were not mapped according to diagnosis, pathology, or medication for participation in the study. There were, however, significant variations in terms of activity levels and experiences with physical activity and exercise in childhood, youth, and early adulthood.

M.H.Ø. recruited the participants by sending an overall request to the general manager/director at the activity center. This was followed by a meeting with the manager and two employees in charge of the exercise sessions, aiming to present and discuss the purpose of the study and ethical considerations. The study sample was selected strategically aiming to gain insight into diverse experiences. The two exercise instructors were asked to choose eligible participants from the activity center, i.e., participants able to answer the aim of the study as best as possible and strengthen the information power and the breadth of variation in the empiricism [60]. The inclusion criteria were having mental health challenges and having participated in the tailored exercise sessions at the activity center over the previous six months or longer. The exclusion criteria were related to researcher safety, where those individuals with psychosis or who were in a major crisis would not be interviewed. Furthermore, it was not desirable to interview participants who could be under the influence of drugs. There was no requirement for physical fitness or functional level. 

All invited participants voluntarily agreed to participate in the study and were then contacted by the M.H.Ø. to arrange the interview. The researcher explained the purpose of the study, assured data confidentiality, and informed the participants about the interview being recorded before starting. All participants completed written informed consent forms.

### 2.6. Data Collection

Data were collected via individual, semi-structured, in-depth interviews [60,61]. A semi-structured interview guide was developed by the M.H.Ø. and K.K.D. and discussed with the research group. The interview guide included open-ended questions formulated to inspire open dialogue, act as a trigger or stimulus for further conversation, and encourage the participants to speak freely about experiences of the adapted/tailored exercise at the activity center. The main questions were; *(1) What made you start exercising? (2) Can you tell me something about your experiences with participating in adapted training here at the activity center? (3) What does exercise mean for your mental health? (4) Have you experienced anything here that has been important to you? (5) Is there anything else you want to tell me?*

All the interviews were carried out by M.H.Ø. at the activity center during the autumn of 2019. The resulting interviews varied in length between 25–45 min. During the interview the interviewer repeated the terms the participants used in the dialog, and various statements were asked again to ensure the interviewer understood correctly what was said. In the end of the interview the interviewer made a summary of what the participants had said, to ensure the interviewer understood it correctly. All interviews were audio-recorded, and the researcher took notes to assist the subsequent analysis. M.H.Ø. and K.K.D. read all the transcribed interviews, which amounted to 78 pages of empirical material.

### 2.7. Data Analysis

The interviews were analyzed using the principles of systematic text condensation [60,62], a modified version of Giorgi’s phenomenological approach [64]. Aiming to create a wider analytic space, the analytical work was conducted as collaborative negotiations between M.H.Ø. and K.K.D. During this discussion, themes and subthemes were gradually developed. The analytic process was characterized as an ongoing and dynamic process, and themes and codes were treated as flexible entities being reconsidered and reorganized several times as new patterns appeared. A stepwise approach as described by Malterud was used [60,62]. The four steps comprised:

(1) Total impression—from chaos to themes

Transcribed interviews were read separately to establish an overview of data and obtain an overall impression/to get a general impression of the whole, looking for preliminary themes associated with participants’ experiences with exercising at the activity center. Preliminary themes were identified and discussed, and browsing the transcripts for the first time, our attention was attracted by themes such as; *“come as you are”, “tailored exercise”, “support from coaches”, “exercise is the best medicine”, “it’s more than just exercise”, “economy”, “the race”, “physical and mental benefits” and “social support”*. Preliminary themes were starting points for organizing data but did not constitute categories or results.

(2) Identifying and sorting meaning units—from themes to codes

Each transcript was reviewed systematically, line by line, to identify meaning units in text, with the themes found in step 1 as road signs. A meaning unit is a text fragment containing some information about the research question [62]. Meaning units containing relevant information of different aspects of the participants’ experiences of the exercise at the activity center were decontextualized and coded thematically into detailed tables of each interview. The coding process included identifying, classifying, and sorting meaning units potentially related to the previously negotiated themes. Identified meaning units were marked with a code—a label that connects related meaning units into a code group. During this process, themes were discussed and consequently renamed and merged based on overlaps, as well as new themes emerging. Codes were re-defined accordingly. During coding, the names and features of the code groups were elaborated from the themes from the first step of the analysis. Following this iterative process, with negotiation, we finally sorted the meaning units according to these codes; *“Inside versus outside”, “Come as you are”, “More than just exercise”, and “Stopover for the way forward”*.

(3) Condensation—from code to meaning

In this step, we systematically abstracted meaning units within each of the code groups established in the second step of the analysis. The meaning units were reread, and with the code groups as starting points, the empirical data were reduced to a decontextualized selection of meaning units sorted thematically (as thematic code groups) across individual participants. A condensed text summarizing the main content in each code group was then developed, and “golden quotes" were chosen.

(4) Synthesizing—from condensation to descriptions and concepts

Finally, the condensed text was converted into an analytical text concerning the participants’ experiences with the exercise, and quotes were given as illustrations.

### 2.8. Ethical Considerations and Procedures

The participants received written and verbal information about the study, and they gave written consent to participate in the study. They were guaranteed anonymity and informed that they could withdraw from the study anytime, without any further consequences. The study was approved by the Norwegian Center for Research Data (ref. 465089). In the study, a qualitative research method was used, and knowledge was created in the interaction between participants and interviewers [61]. Ethical research practice is, however, not just following guidelines and procedures. The interviewer was aware of the asymmetric relationship during the interviews and reflected on their own attitudes and values before meeting a vulnerable group of people [61]. In addition, the interviewer was aware of the nonverbal and verbal language to avoid language power. Furthermore, the place phenomenology was emphasized, as the interviews took place in a room at the activity center, so participants were in a safe and familiar environment [65]. Rooms that are pleasant promote the experience of respect and support in meeting people who are facing challenges [65]. Candles were used, and refreshments served to create a good atmosphere that should inspire an open dialogue. Every effort was made to safeguard the participants’ right to privacy, welfare and care in the best possible way throughout the study. It was important for the interviewer to meet the participants in a friendly, polite and respectful manner, and to have respect for the participants’ perspectives and opinions, even if they were different from her own. This is so that they would feel taken care of in the best possible way as well as showing respect for the time they gave to participate in the study. Attempts have been made to convey the empirical findings with respect and humility for those involved in the study and in a way that is true to the participants’ voices. The authors have been conscious of the language, so that it is the participants’ voices that have been given prominence.

## 3. Findings

**Figure 1:**
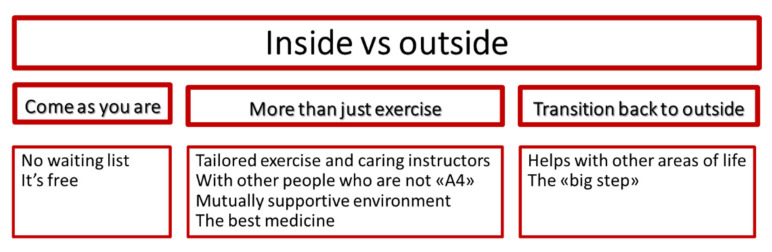
Overarching theme, main themes, and subthemes.

When speaking about their experiences related to exercising at the activity center the participants mostly compared “inside” (referring to inside the activity center) with “outside”, indicating that for them there is a difference between being inside the activity center and outside in the community. Hence, findings were summarized into one overall theme: “Inside vs. outside”. The participants’ experiences with exercising at the activity center were further divided into three main themes: (1) “Come as you are”, (2) “More than just exercise”, and (3) “Transition back to the outside”. A thematic map was created to illustrate these themes (Figure 1). Below the experiences are further presented thematically. Themes and subthemes are formulated based on the participants’ own words, and to be true to the richness and variation in the participants’ descriptions, findings are presented as condensed and detailed descriptions of the experiences. Quotes are provided to illustrate the themes. 

### 3.1. THEME 1: Come as You Are

Most participants valued the activity center as a place where they could “come as they are”. Meaning that for them, the center was a safe space where they did not have to pretend to be anything; they had permission to be vulnerable, flawed and present their physically and mentally natural selves. As one of them expressed it, *“It’s a place where you can just step inside and present yourself.”*

#### 3.1.1. No Waiting List

To enter the activity center, the participants did not have to fill out forms, be interviewed, assessed, or pass through a lot of time-consuming bureaucracy. No referral was needed, and there was no waiting list. These experiences contrasted with their previous experiences of going to new places, which were experienced as being scary, challenging, and time-consuming. One participant described a previous experience by saying,

*“...the first time I got ill there were interviews, forms and a lot of bureaucracy, ...it took time and I got sick. I needed help but didn’t get it, you get forms and assessment just to get into something in the form of help, I thought it was incredibly difficult.”*

For the participants, the bureaucracy in other places was also experienced as if they were being asked to prove themselves worthy or eligible to be offered help.

#### 3.1.2. It Is Free

Several participants talked about their finances and how they appreciated that going to the activity center was "free of charge". Due to their mental health challenges, many participants had experienced a deterioration in their financial situation. Some mentioned that previously they had worked out at regular gyms or participated in other activities but could no longer afford it. As one participant stated, *“I love to be involved in things, but I do not have the finances because of my mental health now.”* At the activity center, finances were no longer an obstacle to participation, they could just “enter and attend”, and even have a personal trainer. One participant said,

*“I work out with my personal trainer (at the activity center) once a week, what if I should pay the money myself, me being disabled. I have the lowest level of insurance, and so it is completely out of the question to spend money on a personal trainer because there isn’t even enough for everyday needs, so it’s not possible no matter how much I need it...”*

### 3.2. THEME 2: More Than Just Exercise

When speaking about their exercise experiences at the activity center, the participants described it as “more than just exercise”, and many experiences contributed to this description.

#### 3.2.1. Tailored Exercise and Caring Instructors

All participants expressed a preference for the tailored exercise at the activity center. The participants described the exercise as always being tailored to their physical fitness and mental state. They valued the competent and facilitating instructors who motivated them and enabled everyone to participate by finding alternative exercises or dividing the group into different levels during the sessions. The aim was for everyone to participate; nevertheless, the participants emphasized that the instructors also respected their personal limits and various (psychological) barriers. Experiencing the activities as approachable and responsive to the diverse needs of the participants were important. As described by one participant,

*“Since the instructors know me so well and they know how far/hard they can push me, and when I’ve been pushed far the instructors pushes me even longer. So, I really like exercising with my personal trainer, because he always knows what I need.”*

The instructors were also perceived as inclusive, non-judgmental, supportive, interested, and caring. When participants were “having bad days” and struggling with motivation, anxiety or mood, they appreciated having regular exercise schedules and appointments. If, however, the participants did not show up, then the coaches sometimes called or asked for them. As one participant said,

*“I believe that for anyone with a mental health challenge, it is difficult to motivate themselves. I got help from the (instructors) here. They called, sent me text messages. So even if I felt it was fussy, it was kind of good because they showed that they cared about me.”*

Being contacted by the staff was experienced as a motivator for participation and regarded as being cared for. At the activity center, they experienced compassion, acceptance, and support. The participants felt welcome, seen, heard and respected, and this was motivating. These experiences contrasted with their previous experiences of feeling alone at regular gyms, where the other attendees were better able to function and were just getting on with things. As articulated by one participant,

*“It’s much harder to just step into an empty gym to exercise without someone telling you what to do and motivate you to exercise, and you don’t quite know what to do inside that gym, then it can be a little overwhelming.”*

Going into an ordinary fitness gym alone seemed overwhelming for this participant, feeling unsure of what to do and where to start. At the activity center, they were exercising in a group with others in the same physical shape, and there was no focus on the body, there were no mirrors, they could not and did not need to look at (their) bodies. Like one said, *“It is movement that is important, and it is a good way of having a safe exercise environment”.* Being part of a group was experienced as a motivator and a driving force to continue exercising for several of the participants. 

#### 3.2.2. With Other People Who Are Not “A4”

All participants indicated positive experiences with exercising together with others “in the same situation”, meaning together with others with mental health challenges. One participant used the metaphor, *“being with other not A4-people”*. The participants experienced being understood by the others at the activity center; they did not have to explain why they “are as they are” or why they are “having a bad day”; they were “all the same”. They appreciated that at the activity center their presence was not dependent on their “daily form,”. At the activity center they were always included, and everyone was “like-minded”, and “nobody is above anyone”. As so aptly described by one participant,

*“It’s a difference between being out there and in here at the activity center, here you can be who you are no matter what; you are understood. It’s really nice to come here to see that other people can have it just like me. That everyone is somehow not perfect out in the world either, ...That people are people.”*

Exercising together with others “in the same situation”, or who had also been through or were in heavy situations (meaning difficult life situations or events), was emphasized as important and positive. One participant said, *“It is one of the best things about this, to exercise with others in the same situation. Because you see that you are not alone with how heavy it is.”* The social environment at the activity center was described as a community without judging. Again, the participants compared it to outside in the community, and it is clear; for them, there was a divide. As one participant expressed, *“I feel there is a little less judgment here. Here it is like; you have got yours and I have got mine, here it’s exercise we’re doing.”* Whereas, outside in society, many participants experienced that if once being labelled as mentally ill, you will never get rid of that label. One participant described it as follows,

*“I feel that you are easily stigmatized in society in general if you are not an A4 person, then you get the label of being weird and mentally ill and all that and then it is not easy to get rid of that label...If you get labelled as mentally ill then it’s forever. It makes it difficult because you are so much more than mentally ill.”*

#### 3.2.3. Mutually Supportive Environment 

Participants also highly valued the mutually supportive environment at the activity center. They experienced receiving support from employees and other participants, and this was perceived as important for recovering. One participant compared it to having another serious illness, *“You can’t get out of this alone. I think it is the same as cancer. One cannot get out of cancer alone.”*

In addition to receiving support, exercising together with others “in the same situation” also represented an opportunity to help and support each other. Showing support for others was experienced as an opportunity to show other sides of themselves. Even though they had difficult days themselves, it was perceived as good to help others. They experienced being able to give and receive "social support for each other”. As one said,

*“You receive support and care, but eventually when you become confident in yourself, you give back and there are important things to experience and learn. Yes, being important to others is also important.”*

Being at the activity center was experienced as being important for others, and the activity center turned out to be a social network, and they made friends. Many of them even used the term “family” when talking about other participants at the activity center. As one said, *“You meet a lot of nice people who do not work here, and I feel everyone here is a small family.”* The feeling of being part of something, belonging and being accepted for who they are, seemed to be important and positively valued. One participant talked about making friends and the feeling of being asked after when ill and not present. To be asked after was described as very big, *“The people at the activity center have even said they have to pick me up, and yes it’s just a trifle, but it can be very big. And that’s the psychosocial for me, I don’t have so much family, so I actually feel quite lonely the other days of the week.”*

#### 3.2.4. The Best Medicine

When speaking about the experiences related to exercise at the activity center, the participants mentioned improvements in both mental and physical health. Some participants even used the words “exercise is the best medicine” when they talked about the progress the exercise gave them. One of them summarized it as follows,

*“I notice it in so much. Attitude, sleep, less thinking, I get better self-confidence, feeling of mastery and attitude, more peace of mind, more energy, you have more stamina, and it feels good to exercise.”*

The experience of exercise energizing them, the rest of the day going easier after having exercise, was a common experience. One participant said,

*“Exercise gives me more structure, just to be at home, you don’t get up in the morning because you have nothing to go to or do, or you feel that you are not good enough for anything.”*

For the participants, the exercise represented something “to go to”, which in turn got their minds and time busy. Many explained how going to exercise resulted in less energy left to “dwell” on negative thoughts. Whereas for others, alcohol and drugs were now replaced by exercise. Like one said, *“There is no longer a need for drinking when bored.”* Now, if the participant was going to do some exercise, there was something to look forward to and something to go to. 

In addition to the psychosocial benefits, exercise also improved their physical fitness. Several participants emphasized how the exercise gave them a stronger body. For someone, increased body strength also gave them a feeling of looking better, and they talked about getting comments from friends and family saying they looked better after they started exercising. Like one participant expressed it,

*“There are many who say that I look better now than before when I started, they see results, and that is always really fun to hear from others, of course. Because then you know that it works and that you develop. Yes, I think that feels good to everyone, really.”*

In the beginning, some participants had experienced exercise as something they "had to" or "should bother to do”. One participant said that he did not really like physical activity. Nevertheless, due to the feeling of being part of something, a group, and the exercise giving strength in the body perceived as important to be able to master everyday life, the participant continued to exercise. After having exercised for a period, some said that they felt that they exercised too much. Then it could be «wrong» that way too. However, they continued to exercise as the advantages outweighed the disadvantages.

### 3.3. THEME 3: Transition Back to Outside

Participants spoke in terms of the activity center as being “inside” and the rest of the world being “outside” and that the activities they were engaging in at the activity center were helping them feel physically and mentally better and more robust, and more able to engage with the “outside”. Taking part in activities provided them with a safe space to heal and build their confidence towards transitioning back to the outside world.

#### 3.3.1. It Helps with Other Areas of Life 

Several of the participants underlined that the experiences they gained from exercise also transferred to other areas in life. One participant said that she could use the coping strategies she had learned in exercise as support in situations outside the activity center. And further expressed, *“It moves to so many other areas in life.”* It was a way to gain enough self-confidence to be able to exercise at ordinary gyms, to go back to work. Some participants articulated that for them, the activity center was an “intermediate station for the way forward”, to get enough confidence and energy to cope in a job and in everyday life. As one participant described it, *“in a way, some of the goal is to get us out to exercise with the “normal”. That we should be able to do it ourselves then and do not need the support around us. But as they (the staff at the activity center) say; “We’re not going to throw you out either”.”* All participants emphasized that the activity center and the sociality they received there was a good form of a self-help strategy. Many of them stated, “Self-help is the best help.”

#### 3.3.2. The Big Step 

Many participants also talked about participating in a local running race being arranged “out there” in the local community every year. Participating in this race was described as “the big step”, and it was taken after much support from the activity center. Participants talked about how they started to prepare both physically and mentally a long time before the race. In advance of the race, it was the unity and the exercise that helped them prepare. Most said they would have never signed up if it was not for being part of a group. After the race, they all expressed a great feeling of mastery; they had accomplished something big and were proud of themselves and the group. Finishing the race was experienced as a great victory. It was described as follows by one participant (being unsure of participating in the race in the beginning),

*“But then I tried it, and it was fun. A huge feeling of mastery. It only gave me a good experience; people were very kind, and they cheered you on. There was nothing scary like I had imagined. You just feel you are being part of a big thing, and you also manage to do it. Yes, that is what is absolutely great, a good feeling.”*

Participating in the race also represented encouragement and motivation for continuing to exercise. The participants consistently talked about setting goals, all the time higher goals. Like one said,

“Yes, you set up goals in advance so you can see that you can do it, reach the goal. It’s so much fun, and it was a victory to get five km, and then I thought I must have 10 km, then it was 10 km, so next time I thought it must be a half marathon.”

Another participant had a desire to run a half marathon again the next year and even talked about another and higher goal, “Next year there is a half marathon again, but my wish is great: I want to run a marathon. Yes, when the body can tolerate it. My goal now is actually to double the running length.”

## 4. Discussion

The purpose of this qualitative study was to explore how individuals facing mental health challenges experience participating in tailored exercise at a community-based activity center, and what role tailored exercise could play in supporting an individual’s mental health recovery. The participants described a view of their experience at the activity center and the rest of the world as an inside versus outside dichotomy. Inside was a safe space where they felt accepted, seen, and heard as individuals, supported according to their needs, and respected and valued as human beings. The outside world was “normality” and represented a state and a social environment that was currently not comfortable or sometimes even felt cold and hostile. According to our findings, the community center was experienced by the participants as a liminal space. Liminality refers to moments or periods of transition during which normal limits of thought, self-understanding and behavior are relaxed, opening the way to novelty and imagination, construction and destruction [66,67]. The community center as a liminal space created an environment where normal limits of thought and behavior were relaxed, and people could present themselves as they were, even on bad days. They felt they did not have to expend energy concealing their true emotions, their financial, physical, or mental struggles, and they could be accepted as they were. This experience opened them to self-acceptance, feeling validated and valued, and being able to engage with exercise, which they often gave up in “normal” gym environments. 

The intention of the activity center is to support the participants in their recovery process. Our results provide insights into what role the tailored exercise at the activity center could play in supporting the participant’s process of recovery. The described dichotomy might, however, point in the direction where citizenship becomes the ultimate goal, maybe more than “mental health recovery”, which can be understood as living well with mental health problems. Several findings deserve highlighting. 

First, participants emphasized the importance of being able to come as they are at the activity center, without being interviewed, measured, or subjected to bureaucracy. In addition, entering the activity center was free of charge; hence their financial situation was no longer an obstacle to participation in activities together with others. The Norwegian Government aims to even out the increasing social inequalities in health [19], and within a recovery-perspective, seeks for all participants to have the opportunity to live active lives, emphasizing participation in meaningful activities in a good social community, where they can be themselves [2,43]. 

Participants also described exercising in a group with other individuals facing mental health challenges and being introduced to tailored activities and instructors specialized in physical activity and health as important for their participation in the activities. These experiences are in accordance with qualitative studies on physical activity among people facing mental health challenges; therefore, these findings should not be surprising. Embedded in this are, however, also the aspects of connection and community. As it appears in our data and other qualitative studies, the exercise allowed them to connect with others [23,25,27,28,45,46,47,48,49,50]. In addition, several of the participants’ descriptions naturally comprise what we here consider as affiliation and construction of positive and “collective identity” [41,68,69]. We suggest that experience of affiliation might even be the most important ingredient of the exercise at the activity center. Collaborations, relationships, and peers are significant contributors to increase hope [44], and recovery is dependent on a sense of belonging [5]. Considering how the participants in this study described how if they had a bad day, it was not a problem. The positive thing about someone making contact and asking for you is that it shows that you are a significant member of the group. This says something about the human vision and culture among the participants. The term “family” when talking about other participants at the activity center, and to be asked after when not present, might, however, be an expression of community and connection, which are needs common to all humans. The participants experienced that they made friends and a social network at the activity center, and connectedness is a key part of an individual’s personal recovery process [5,42,44]. 

The subtheme “With other people who are not “A4”” and the common experience of facing mental health challenges and be stigmatized also represents a form of “collective identity”, as “ill”, but with hope of getting well as something likely to happen. Inside the activity center, they could be themselves; it was perceived as an inclusive and non-judgmental environment. This contrasted with participants’ previous experiences of the stigmatization of people with mental health challenges outside, in society. They experienced it as challenging, being in a recovery process outside in the society, as the network and people “out there” did not understand that being mentally ill does not mean you cannot change and recover [15], or constantly being reminded that you are «mentally ill» and hence not an «A4 person». 

The descriptions of “being happy” at the activity center might be an expression of hope, meaning and optimism for the future, which, together with connectedness/belonging and identity, are considered important for a recovery process [5,15,42,44]. These experiences contrasted their experienced feelings of “not being good enough for anything” when just staying at home. In line with previous qualitative research [23,24,25,27,28,45,46,48,50,51], the participants in our study experienced the exercise as having something to go to, something to look forward to, something meaningful to fill their days with, as well as providing a structure. 

The participants in the present study also highlighted “the mutually supportive environment” at the activity center, including both receiving and giving support from employees and other participants. This notion that mutuality in relationships has a great value and importance has been noted in much research and theory. As noted above, within the CHIME framework both connectedness to other people and the notion of having socially valued roles (being the helper) underpins such ideas [42,44]. These themes are also found in the review by Dell, Long [5] and outlines the importance of mutuality in relationships as an important aspect of recovery. This is related to research on the concept of mattering [70]. Being cared for can be understood as feeling valued, but being able to give care can be understood as adding value. Prilleltensky [70] has shown how the concept of mattering correlates to the feeling of well-being, both on individual levels and on the societal level. The research literature within the different domains would probably all support such a notion. Research from the Harvard Study of Adult Development may add some further weight to the extent that relationships have a profound impact on our general health and well-being [71].

In accordance with previous research [23,25,53,54], this study also demonstrates the central importance of facilitating personnel, and that the experience of supportiveness, respect, and caring might be the most important ingredients. The experience of the instructors calling for them if they did not show up was experienced as the instructors doing something extra beyond what was expected. In user stories about what is experienced as help to “recover”, the personnel who sees the person and does the little extra beyond the participants’ expectations are often highlighted as a support for the participants in their recovery process [2,3,16,72]. 

It is interesting to note that the participants in the current study described exercise as “the best medicine” due to the experienced mental improvements and symptom relief following exercise, which is also frequently reported in research on physical activity for people with mental health challenges [23,24,25,26,27,28]. Often, recovery as a personal process is distinguished from recovery as a clinical process. However, it is argued that it does not have to be an either/or question, the clinical recovery can be an important part of personal recovery [5]. 

A surprising and important finding in the current study was that the participants experienced the activity center as an “intermediate station for the way forward” until they got enough self-confidence to be able to exercise at an ordinary fitness center and go back to school or jobs. However, as the staff at the activity center stated, *“We’re not going to throw you out either”*, which again highlights affiliation and empowerment of the participants. It was up to the participants themselves for how long they wanted to take part in the activities; participation was not limited in time. As also reported in previous research [26,46,51,52], the results from our study indicate that the participants experienced participating in tailored exercise at the activity center together with others in the same situation as a good “self-help” strategy.

However, our results do not reveal what happens to the participants and their recovery process when they are no longer exercising at the activity center. Whether participation in exercise continues to contribute to their recovery process when they are no longer participants at the activity center is yet to be confirmed. Nevertheless, as the study reflects, the tailored exercise offers more than just exercise. It offers a liminal space where the participants may develop the needed skills and confidence to take part more fully in society, and as such, develop what the literature defines as citizenship [73]. Whether the participants choose to pursue physical activities outside the center may not be the most important aspect. A more fruitful question may rather be whether the participant somehow can utilize their experience through the liminal space to further develop their citizenship. There is a growing body of research within the field of citizenship, and other adjacent fields of research such as “well-being”, “relational welfare”, “mattering” and the Act-Belong-Commit campaign (ABC) that underpins this important concept of participation in society as a means to mental health recovery [5,70,74,75]. 

Reports from the participants in our study support these kinds of ideas and add knowledge to our understanding of how such processes of recovery and citizenship may be facilitated and supported through our ability to create facilitating liminal spaces. This research shows that tailored physical exercise in a community-based activity center may be just such a liminal space that can facilitate mental health recovery and citizenship. It is important to recognize that people have an innate desire to recover, to rehabilitate themselves, and they recognize the value of structure and movement. They want to engage with the “normal” world to experience citizenship, but need a safe transition space to do this effectively. 

### Methodological Considerations 

The study reported in this paper adds to the existing literature an in-depth qualitative analysis of experiences of participating in tailored exercise at a community-based activity center for people facing mental health challenges and identifies a dichotomy between inside the center and the outside world, where the activity center acts as a liminal space that builds people’s capacity to make the transition back to normality. Participants were all from one geographic area in the south of Norway, most participants in this study were white (representative of locality), and interviews in other locations or with more diverse groups of people may have produced different information. The interpretation of the interview data presented in this paper is only one of many possible interpretations. To ensure that the interpretation was a fair representation of the interviewees’ views and that data analysis decisions were transparent, a rigorous process was adopted in which data were double-coded by the researchers, and disagreements were resolved in team discussions throughout the coding process. Moreover, a detailed description of all the steps of the research process, from data collection and analysis to presentation of findings, have been provided. Findings from a qualitative study cannot be generalized in a statistical sense for the population of people facing mental health challenges as a whole. Still, the findings can be considered as descriptions that can be used in a specified setting, and due to the detailed nature of the description, it seems reasonable to consider the findings in relation to similar contexts in other subpopulations of people facing mental health challenges. 

## 5. Conclusions

The current study explored how individuals facing mental health challenges experienced participating in tailored exercise at a community-based activity center, and what role tailored exercise could play in supporting an individual’s process of recovery. Our findings revealed that the center acted as a liminal space that aided mental health recovery by allowing the participants to feel safe, accepted and supported, as well as experience citizenship. Regarding the implications for practice, our results support the importance of physical activity as part of mental health work. In addition, the findings highlight and confirm the need for a change in focus to treat mental health challenges as a contextual phenomenon, and service development should add ideas of citizenship as important and an integral part of the service. Future research on physical exercise and mental health ought to study the way physical activity can lead to more citizenship. Creating a liminal space is viewed as a recovery supporting approach, through facilitating an opportunity for recovery. Future research should also explore the ingredients in such a liminal space and common denominators compared to other liminal spaces. Furthermore, through mixed methods, future research should investigate how tailored exercise at a community-based activity center understood as a liminal space leading to recovery and citizenship can influence, enhance and sustain physical fitness and mental health well-being. As a final remark, the corona pandemic has revealed how vulnerable we people are when our community with others is limited and we are being deprived of these liminal spaces. Nevertheless, it seems more attention has been given to the need for more therapy rooms rather than creating arenas for physical activity, community, and citizenship in society.
